# Designing a Sustainable Noise Mapping System Based on Citizen Scientists Smartphone Sensor Data

**DOI:** 10.1371/journal.pone.0161835

**Published:** 2016-09-14

**Authors:** Eunyoung Shim, Dohyeong Kim, Hyekyung Woo, Youngtae Cho

**Affiliations:** 1 Department of Public Health Science, School of Public Health, Seoul National University, Seoul, South Korea; 2 Institute of Health and Environment, Seoul National University, Seoul, South Korea; 3 School of Economic, Political and Policy Sciences, University of Texas at Dallas, Dallas, Texas, United States of America; 4 Department of New Business, Samsung Fire and Marine Insurance, Seoul, South Korea; University of Bologna, ITALY

## Abstract

In this study, we attempted to assess the feasibility of collecting population health data via mobile devices. Specifically, we constructed noise maps based on sound information monitored by individuals’ smartphones. We designed a sustainable way of creating noise maps that can overcome the shortcomings of existing station-based noise-monitoring systems. Three hundred and nine Seoul residents aged 20–49 years who used Android-based smartphones were recruited, and the subjects installed a special application that we developed for this study. This application collected information on sound and geographical location every 10 min for 7 days. Using GIS, we were able to construct various types of noise maps of Seoul (e.g., daytime/nighttime and weekdays/weekends) using the information on sound and geographical location obtained via the users’ smartphones. Despite the public health importance of noise management, a number of countries and cities lack a sustainable system to monitor noise. This pilot study showed the possibility of using the smartphones of citizen scientists as an economical and sustainable way of monitoring noise, particularly in an urban context in developing countries.

## Introduction

The physical environment is an important determinant of population health. Unlike social or biological determinants, the physical environment cannot be overcome or improved by individuals, and its contamination is difficult to reverse. Thus, individuals are often exposed to harmful environments, and accurate and continuous monitoring of the physical environment is vital. This may be done in various ways depending on the environmental factors of concern, including via monitoring networks, sampling, and image analysis. Monitoring networks are the most common approach in many countries. However, this method is subject to a number of limitations in its coverage and accuracy, particularly when monitoring sites are few or sparsely located, which can result in broad-scale measurements depending on estimates rather than direct monitoring. In addition, when the pollutant discharge changes frequently, it is even harder to monitor and to provide accurate information to exposed populations.

Smartphone penetration has increased dramatically worldwide in recent years. Furthermore, time spent using smartphones has also increased enormously, with charging often required more than once a day. Smartphones are equipped with various imbedded sensors capable of monitoring sound, light, proximity, magnetism, motion, geographic location, and others. In the late 2000s, these sensors were only available to technicians. All smartphone users now enjoy the various functions of their devices, which are facilitated by these imbedded sensors. This, in turn, allows ‘crowd sourcing,’ wherein data sensed by the smartphones of anonymous users can provide copious and valuable information. Crowd sourcing is an inexpensive, effective, and efficient means of data collection that overcomes temporal and spatial boundaries [1, 2]. Debate continues over whether data collected anonymously is suitable for use in, for example, scientific studies or policy development. However, the interest in crowd sourced data collected from devices used by lay people in their daily lives (e.g., smartphones) has gradually increased worldwide, opening the door to ‘citizen science’ or ‘participatory sensing’ [3–7].

Noise is a typical pollutant of urban environments, and the population exposed to serious daily noise has grown rapidly worldwide in recent years [8, 9]. Several attempts have been made to establish noise monitoring maps (or simply noise maps) using data from smartphone sound sensors. Examples include the Noisetube Project [10], NoiseSpy [11], Ear-Phone [12]. These examples illustrate the potential for noise mapping through crowd sourcing and citizen scientist participation. However, most of these studies were able to gather data only when citizen scientists actively monitored and submitted sound levels to a server. This is not sustainable, because active measurements could disturb the daily lives of users, and large-scale mapping would be almost impossible without clear directions. Therefore, the purpose of this study was to explore whether sustainable noise mapping is possible through a crowd sourcing method that acquires information via smartphone sound and geographical sensors while not intruding on the smartphone users’ daily lives.

## Methods

We conducted a pilot study of citizen scientist-based noise mapping in Seoul, South Korea. We recruited 336 adult residents of Seoul, aged in their 20s to 40s, using Samsung smartphones (Galaxy S3, Note 2, and Note 3 models, in which the same microphone is installed) to ensure standardization of the sound sensor. Recruitment and management of the study participants were conducted by Gallup Korea, one of the leading social survey companies in South Korea and a Gallup International branch. Gallup Korea has maintained a social survey respondent group that represents the adult residents of Seoul. They attempted to recruit participants for this study only from their respondent group. However, a number of participants in their respondent group did not have the aforementioned specific smartphones. Gallup Korea thus extended their recruitment to a respondent group living outside of Seoul but who commuted to Seoul daily. Therefore, the participants in the current study physically cover a wide geographic range in Seoul in terms of their residences and/or daily activities.

Participants were asked to install an Android-based application that we developed for this study. This application automatically recorded geographical (longitude/latitude) and sound information to the smartphone every 10 min for 1 week. The sound information was collected through the imbedded microphone. When the microphone was in use for any activities (e.g., telephone or game play), the application automatically surrendered priority to those activities, so as not to interfere with the users’ daily lives. The application also automatically turned the sound recorded by the microphone into a decibel (dBA) value and immediately erased the original sound to protect privacy. The application recorded sound within the range of 0 and 90 dBA. Sound louder than 90 dBA was recorded as 90 dBA because it already reflected a potentially dangerous noise level. The data collection period was 11–25 March 2013. During this period, the participants could choose seven consecutive dates of their own convenience for recording, and all information on sound (in decibels only) and geographical information was accumulated in their smartphones. After 7 days, the application prompted a pop-up on the smartphone, informing the users of the study purpose and procedures again and asking whether they agree to having sensor information sent to the server. To ensure high-quality privacy protection, we arranged for our server to be kept at Statistics Korea (South Korea National Statistics Office), and this organization undertook the initial data screening. Then, Statistics Korea made the data available to the current authors in a form in which no personal information was identifiable.

Since the application gathered sensor information every 10 min for a week, there were supposed to be 1,008 data readings for each participant. However, owing to the nature of the aforementioned surrendering of priority, there were a number of missing values per participant. We treated these values as missing instead of inputted values. During the period of study participation, some participants’ data reading was not performed for days since their smartphone was switched off. We discarded the data from the participants for whom readings were missing for more than 4 days. Thus, a total of 249,713 data readings from 309 participants were utilized in this study.

To construct a noise map that covers the whole of Seoul every 10 min, we first spread the data readings on the map and interpolated the empty space employing the Inverse Distance Weighted (IDW) method, which assigns weights variably by distance from the data spot and is frequently used for environmental monitoring (e.g., air pollution or temperature) and meteorological monitoring [13–15]. The algorithm and basic rules of IDW can be found elsewhere [16, 17].

### Ethics statement

The Institutional Review Board of Seoul National University exempted our research from the need for an ethics review (No.: 3-2013-2-26).

## Results

**Fig 1:**
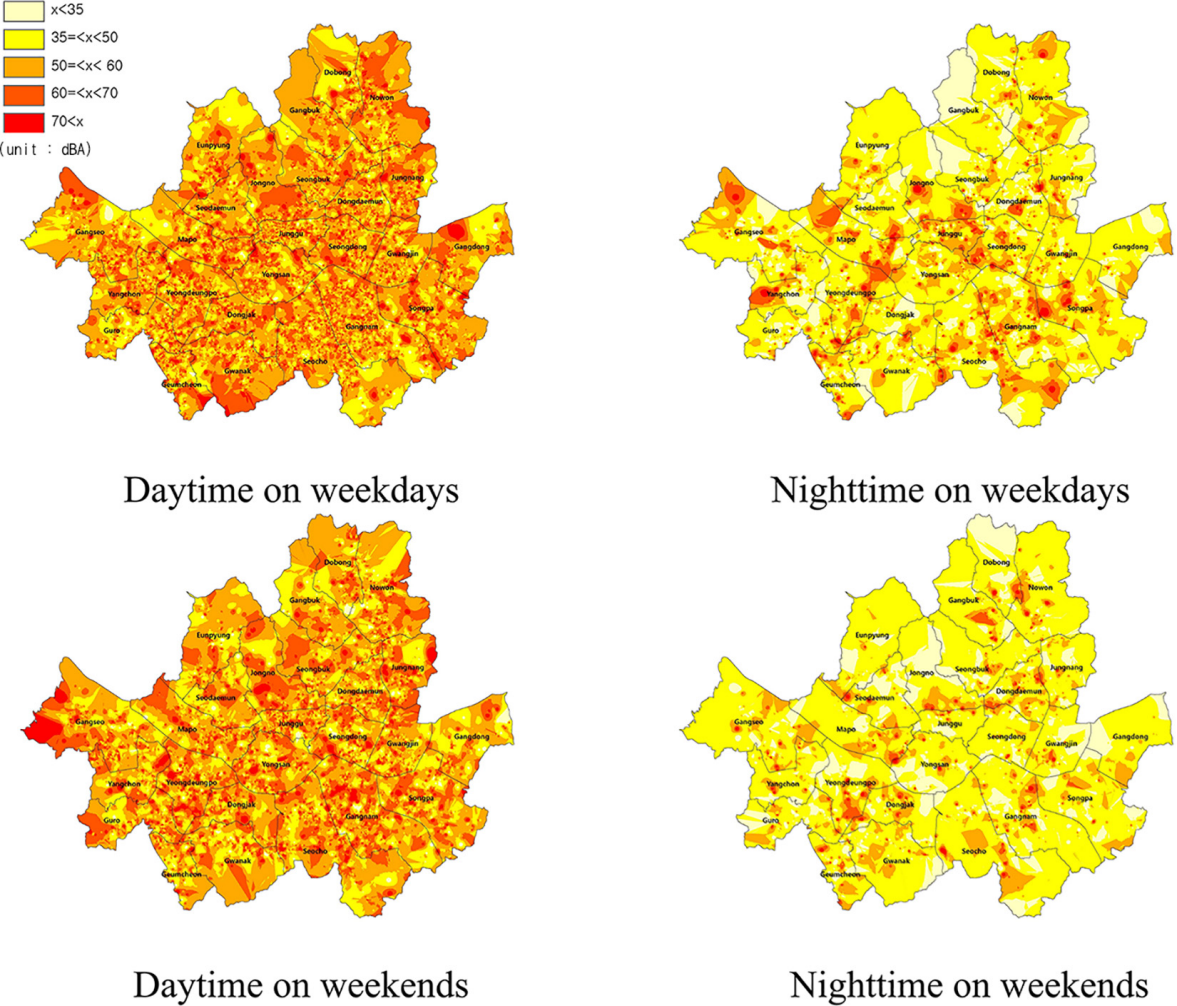
Collective noise map of Seoul, South Korea.

Fig 1 shows snapshot examples of a noise map of Seoul for daytime (06:00–21:59) and nighttime (22:00–05:59) periods on weekdays/weekends. Color represents the average sound value in decibels recorded from corresponding times and geographic locations, and the value of non-recorded space was interpolated by IDW. Daytime was obviously much noisier than nighttime on both weekdays and weekends but, at night, weekdays were noisier than weekends. We can easily see that some areas in Seoul had a noise level much higher than 35 dBA, which the World Health Organization has recommended to avoid at night due to the possible risk of insomnia upon chronic exposure [9]. Because geographical and sound sensor information was collected every 10 min for 1 week, we were able to construct various noise maps (e.g., by time, day, area, etc.). Furthermore, by assuming that geographic sensor information recorded *only* by a wifi-positioning system (WPS) represented an indoor location, we were even able to construct indoor/outdoor noise maps.

## Discussion

Seoul Metropolitan Government’s noise monitoring network has only one monitoring station in each of its 25 districts. Currently, only nine monitoring stations provide real-time noise level information to Seoul’s citizens, while the noise levels recorded by other monitoring stations have been provided with a 9-month delay. It is also unrealistic to expect that a single monitoring station could cover the entire area in which over 400,000 people live (24 km^2^ per district, on average). Therefore, the noise information provided to citizens is just a daily average value in decibels, rather than in the form of a map. The Seoul noise map that we present in this study using smartphone sensor data from 309 study participants contains much more detailed information than the current noise monitoring produced by Seoul Metropolitan Government. For various reasons, including budget and the location of monitoring stations, it is almost impossible for the government to expand the noise monitoring network to a level sufficient for establishing a noise map. Seoul may not be the only metropolitan city that lacks a meaningful and useful noise monitoring system, and the situation does not appear to be better in most large cities globally, particularly in developing countries. Therefore, noise monitoring by citizen scientists using smartphone sensors is a realistic and sustainable alternative, which is timely, efficient, economical, and environmentally friendly.

To establish an effective noise monitoring system, several hurdles need to be overcome. The first is related to privacy. Since GPS information is a key element of such systems, it may not be easy to recruit citizen scientists who are willing to disclose their location every 10 min, irrespective of the level of security (e.g., encrypted storage without any personal information). A possible solution to this is to maximize the anonymity of the participants by increasing the number recruited, while decreasing the frequency of sensor recordings. For instance, in the current study, we included approximately 300 participants who provided sensor information every 10 min for 7 days. The study thus comprised 302,400 records. The same number could be achieved by data readings only once an hour if the number of participants were 1,800. The second issue is the accuracy of the noise measurement by smartphones. Recent studies have verified that smartphone sound sensors measure sound as accurately as professional noise meters [18–21]. However, despite this accuracy, the measurement of noise using a smartphone sensor can be seriously affected by how users carry and use their smartphones and by the location of the smartphone (altitude, vibration, wind, air pressure, etc.), which may result in inaccurate results. For example, if a smartphone were located in a pocket or a purse, it could not accurately measure the actual ambient noise level. If one hopes to establish a noise mapping system that accurately measures the noise level, the method presented in the current study would not suffice. However, the method employed here is sufficiently economical, sustainable, and efficient for most purposes, particularly for public health and environmental monitoring. Nonetheless, future studies should develop approaches to adjust various factors by incorporating other smartphone sensor data, such as using a gravity acceleration sensor and an air pressure sensor [22–24].

### Public health implications

There are still issues to overcome, including the accuracy of smartphone sound sensors, standardization, and citizen scientist recruitment. Nevertheless, we believe that the noise mapping presented here has significant merit even in its current form, particularly in the urban context of developing countries where traffic and construction work are common, and noise monitoring systems absent. A dense, real-time noise monitoring system will benefit both participants and non-participants by live-logging daily noise exposure as a form of personal health record. Furthermore, the accumulation of noise data for a period much longer than 1 week, as applied in the current pilot study, would provide big data that can be linked to other data sources, such as health insurance data or census data, enabling us to develop new knowledge on health and various aspects of society.
